# Comparison of manual and automated fiber quantification tractography in patients with temporal lobe epilepsy

**DOI:** 10.1016/j.nicl.2019.102024

**Published:** 2019-10-17

**Authors:** Barbara A.K. Kreilkamp, Lucy Lisanti, G. Russell Glenn, Udo C. Wieshmann, Kumar Das, Anthony G. Marson, Simon S. Keller

**Affiliations:** a Department of Molecular and Clinical Pharmacology, Institute of Translational Medicine, University of Liverpool, Liverpool, United Kingdom; b Department of Neurology, The Walton Centre NHS Foundation Trust, Liverpool, United Kingdom; c Royal Society, London, United Kingdom; d Department of Radiology and Imaging Sciences, Emory University, Atlanta, GA, United States; e Department of Neuroradiology, The Walton Centre NHS Foundation Trust, Liverpool, United Kingdom

**Keywords:** Automated tractography, Manual tractography, Hippocampal sclerosis, Surgical outcomes, Temporal Lobe Epilepsy

## Abstract

**Objective:**

To investigate the agreement between manually and automatically generated tracts from diffusion tensor imaging (DTI) in patients with temporal lobe epilepsy (TLE). Whole and along-the-tract diffusivity metrics and correlations with patient clinical characteristics were analyzed with respect to tractography approach.

**Methods:**

We recruited 40 healthy controls and 24 patients with TLE who underwent conventional T1-weighted imaging and 60-direction DTI. An automated (Automated Fiber Quantification, AFQ) and manual (TrackVis) deterministic tractography approach was used to identify the uncinate fasciculus (UF) and parahippocampal white matter bundle (PHWM). Tract diffusion scalar metrics were analyzed with respect to agreement across automated and manual approaches (Dice Coefficient and Spearman correlations), to side of onset of epilepsy and patient clinical characteristics, including duration of epilepsy, age of onset and presence of hippocampal sclerosis.

**Results:**

Across approaches the analysis of tract morphology similarity revealed Dice coefficients at moderate to good agreement (0.54 - 0.6) and significant correlations between diffusion values (Spearman's Rho=0.4–0.9). However, within bilateral PHWM, AFQ yielded significantly lower FA (left: *Z* = 4.4, *p*<0.001; right: *Z* = 5.1, *p*<0.001) and higher MD values (left: *Z*=-4.7, *p*<0.001; right: *Z*=-3.7, *p*<0.001) compared to the manual approach. Whole tract DTI metrics determined using AFQ were significantly correlated with patient characteristics, including age of epilepsy onset in FA (*R* = 0.6, *p* = 0.02) and MD of the ipsilateral PHWM (*R*=-0.6, *p* = 0.02), while duration of epilepsy corrected for age correlated with MD in ipsilateral PHWM (*R* = 0.7, *p*<0.01). Correlations between clinical metrics and diffusion values extracted using the manual whole tract technique did not survive correction for multiple comparisons. Both manual and automated along-the-tract analyses demonstrated significant correlations with patient clinical characteristics such as age of onset and epilepsy duration. The strongest and most widespread localized ipsi- and contralateral diffusivity alterations were observed in patients with left TLE and patients with HS compared to controls, while patients with right TLE and patients without HS did not show these strong effects.

**Conclusions:**

Manual and AFQ tractography approaches revealed significant correlations in the reconstruction of tract morphology and extracted whole and along-tract diffusivity values. However, as non-identical methods they differed in the respective yield of significant results across clinical correlations and group-wise statistics. Given the absence of excellent agreement between manual and AFQ techniques as demonstrated in the present study, caution should be considered when using AFQ particularly when used without reference to benchmark manual measures.

## Highlights

• Tractography approaches showed moderate to good agreement for tract morphology.
• Along- and whole-tract diffusivity was significantly correlated across approaches.
• Whole-tract AFQ but not manual tract diffusivity correlated with clinical variables.
• Absence of excellent agreement between approaches warrants caution.

## 1 Introduction

Temporal lobe epilepsy (TLE) is a brain disorder characterized by widespread white matter (WM) and gray matter (GM) changes, which extend throughout the brain (Bernhardt et al., 2013; Keller and Roberts, 2008; Richardson, 2012; Bonilha and Keller, 2015; Rodríguez-Cruces and Concha, 2015; Gross, 2011). Extensive brain changes like these have given rise to the notion that TLE may be considered a brain network disorder (Berg and Scheffer, 2011). Quantitative analysis can extend conventional visual assessment of MRI and is especially useful when modeling network connections in the brain. This can be achieved by using tractography analysis approaches, which shed light on neuroanatomical structure, and may be particularly useful for investigating epileptogenic WM connections. Typically, this is performed using manual tractography, however recently tools that automatically reconstruct WM tract fiber bundles have been developed (Yendiki et al., 2011; Yeatman et al., 2012; Hagler et al., 2009). Automated approaches are more time and resource efficient compared to manual approaches as they do not require time-consuming delineation of tracts by trained researchers (Hagler et al., 2009).

Automated Fiber Quantification (AFQ; https://github.com/jyeatman/AFQ) is a very popular and commonly used automated approach, which permits the investigation of WM tract diffusion alterations between subject cohorts in T1-weighted (T1w) space (Yeatman et al., 2012). It has been predominantly used to assess diffusivity changes along well-defined WM pathways, which is useful for the study of network disorders known to affect local WM connections differently. Diffusion characteristics have been shown to vary along tracts in healthy individuals (Johnson et al., 2013), in preterm children and adolescents (Travis et al., 2015a; Travis et al., 2016), in patients with anorexia nervosa (Travis et al., 2015b), autism spectrum disorder (Libero et al., 2015), depression (Sacchet et al., 2014a; Sacchet et al., 2014b) and in patients with TLE (Keller et al., 2017; Glenn et al., 2016; Concha et al., 2012). In patients with TLE, these localized changes may be related to the severity and chronicity of the disorder rather than to an initial precipitating injury resulting in hippocampal sclerosis (HS; Kreilkamp et al., 2017).

Despite the recent proliferation of studies employing automated tract reconstruction methods, there are no studies comprehensively examining the consistency of results between AFQ and manual tractography approaches with respect to Dice Coefficients, volume differences, whole tract and along-the-tract measurements and group statistical results. Manual tractography may be considered for benchmarked comparisons as it is reproducible across operators by the use of an established protocol (Wakana et al., 2007; Yendiki et al., 2011). Yeatman et al. (2012) correlated fractional anisotropy (FA) diffusion values obtained from manual tractography versus AFQ and reported high Pearson correlations with manual measurements. Across both approaches, Hua et al. (2008) reported FA Pearson correlation coefficients greater than 0.82 for all tract investigated except for the uncinate fasciculus (UF, *r* = 0.52) and superior longitudinal fasciculus, while Hagler et al. (2012) reported Pearson correlation values at >0.9 for all tracts and reduced FA for the automated approach. No study has compared AFQ to manual tractography performed in native diffusion space with respect to whole tract morphology and anatomical accuracy, the estimation of diffusion scalar metrics from tracts and the consistency of results in group comparison studies. Comparisons between manual and automated MRI techniques have frequently been performed in quantitative WM (Zijdenbos et al., 1994) and GM analysis (Keller et al., 2012a). The consistency between manually and automatically delineated brain regions can be investigated using the Dice coefficient (Dice et al. 1945). It is important to develop automated whole and along-the-tract diffusion tractography methods (Concha et al., 2012; Glenn et al., 2016; Keller et al., 2017; Kreilkamp et al., 2017) and a comparison to benchmark manual approaches is needed. Automated techniques provide substantial benefits as they circumvent the need for extensive training of researchers to perform manual measurements and provide reproducible algorithms that can be applied to multiple datasets in an efficient manner. Recent literature has relied on these novel automated methods even though they have not been extensively validated in healthy controls and patients with brain disorders apart from a few exceptions (such as TrActs Constrained by UnderLying Anatomy, Yendiki et al., 2011).

In the absence of histological data, manual techniques are considered to be closest to the ground truth and used as a benchmark in MRI studies. In particular, due to interactive qualitative assessment of the segmented tracts, a trained and expert neuroanatomist is able to avoid and correct any reconstruction problems arising from artifactual subject variability. Fully automated methods do not include the option of interactively and visually checking whether or not individual streamlines are part of a certain tract for fine-tuning anatomical segmentations. Still, their algorithms offer the possibility of avoiding systematic bias that may otherwise occur during manual pruning of tracts. As AFQ is a widely used and successful approach and TLE a common neurological condition associated with structural abnormalities along WM pathways (Müller et al., 2006; Liu et al., 2012), we investigate whether both deterministic tractography approaches (AFQ and manual) are sensitive enough to find these abnormalities. Keller et al. (2017) used AFQ to study three principle white matter tracts in patients with refractory TLE who underwent temporal lobe surgery. Along-the-tract analyses revealed that diffusion metrics of these tracts could predict postoperative seizure outcome with a sensitivity of 84% and specificity of 89%. We analyzed two principle temporal lobe white matter tracts: the para-hippocampal white matter bundle (PHWM; Ahmadi et al., 2009, Keller et al., 2012b) and UF (Lin et al., 2008; Ahmadi et al., 2009) due to their importance in TLE (Keller et al., 2017).

The objective of the present study was to investigate the agreement between manually and automatically generated temporal lobe WM tracts (UF and PHWM) in patients with TLE. We sought to investigate the consistency between the automated and manual approaches with respect to (i) tract morphological characteristics using Dice Coefficient analyses, (ii) similarities in diffusivity scalar metrics (FA and mean diffusivity, MD) and (iii) the sensitivity of each approach for the detection of tract diffusivity abnormalities in patients relative to controls and correlations between clinical characteristics and diffusion metrics.

## 2 Materials and methods

### 2.1 Participants

Demographic and clinical characteristics of patients and controls are provided in Table 1. All patients had electrophysiological evidence of either right- or left-sided TLE, supported by unilateral EEG temporal lobe discharges and seizure semiology consistent with a unilateral temporal lobe seizure onset. A history of brain infection was positive if the patient reported meningitis or encephalitis. Seizure burden was calculated as log(duration of epilepsy x seizure frequency per week).

**Table 1:** Demographic and clinical information for all participants.

	Left TLE	Right TLE	Controls	Stats
N	16	8	40	
Sex (female/male)	10/6	6/2	23/17	χ²(2)=0.9, *p* = 0.6
Mean age in years (SD), Range	32.1 (11.5), 18–61	31.8 (12.3), 19–54	32.4 (8.7), 22–60	χ²(2,63)=0.3, *p* = 0.9
History of infection (yes/no)	0/16	4/8	–	χ²(1)=3.8, p_(Yate)_=0.051
History of febrile seizures (yes/no)	4/12	1/7	–	χ²(1)=0.03, p_(Yate)_=0.9
SGTCS (yes/no)	9/7	5/3	–	χ²(1)=0.02, p_(Yate)_=0.9
Hippocampal Sclerosis (yes/no)	5/11	3/5	–	χ²(1)=0.02, p_(Yate)_=0.8
Mean age of onset (SD)	15.8 (11.4)	18.1 (11.6)	–	*Z*=-0.46, *p* = 0.7
Mean seizure burden (SD)	1.4 (0.5)	1.3 (0.7)	–	*Z* = 0.28, *p* = 0.8
Mean duration corrected for age (SD)	0.5 (0.3)	0.4 (0.3)	–	*Z* = 0.89, *p* = 0.4
Mean seizure frequency per week (SD)	4.9 (8.9)	3.4 (4.7)	–	*Z* = 0.22, *p* = 0.8

TLE = Temporal Lobe Epilepsy; SD = Standard Deviation; SGTCS = Secondary-Generalized-Tonic-Clonic-Seizure.

### 2.2 Data acquisition

We acquired 3D T1w fast-spin-gradient (FSPGR) images with Phased Array Uniformity Enhancement (PURE) signal inhomogeneity correction (140 slices, TR=8.2 ms, TI=450 ms, TE=3.22 ms, flip angle=12, with 1 mm isotropic voxel size, acquisition time: 3:48 mins) for all participants. We also acquired 3D T2w CUBE images (with PURE correction, 312 slices, TR=2500 ms, TI = *N*/A, TE=71.2 ms, flip angle=90, with 0.5 mm isotropic voxel size) and 3D sagittal CUBE T2 fluid-attenuated inversion recovery (T2FLAIR) with PURE (312 slices, TR=6000 ms, TI=50 ms, TE=127.1 ms, flip angle=90 with 0.5 mm isotropic voxel size). Acquisition time was 3:18 min for the T2w image and 7:27 min for the T2FLAIR CUBE image. DTI data were acquired using a 60-direction spin echo pulse sequence (66 slices, TR = 8000 ms, TI = *N*/A, TE = 82 ms, flip angle = 90, voxel size = 1 mm x 1 mm x 2 mm, no cardiac gating, with ASSET, b-value = 1000s/mm2; FOV = 256 mm) with six b0 images without diffusion weighting. The acquisition time was 8:56 min. We also acquired coronal T1-FLAIR (52 slices, TR = N/A, TI = 920 ms, TE = 9.94 ms, flip angle = 111, voxel size = 0.4 mm x 0.4 mm x 3 mm, acquisition time 4:00 min) and T2-FLAIR (40 slices, TR = 12,000 ms, TI = 2713 ms, TE = 98.7 ms, flip angle = 160, voxel size = 0.86 mm x 0.86 mm x 4 mm, acquisition time 3:24 min) sequences for diagnostic and incidental finding purposes in all participants. All images were assessed by expert neuroradiologists.

### 2.3 Data preprocessing and quality assurance

Before tensor fitting and tractography were performed, DTI data was processed using FMRIB Software Library (FSL) version 5.0.9 (http://fsl.fmrib.ox.ac.uk/fsl/fslwiki/FSL) according to the ENIGMA DTI-preprocessing steps to mitigate effects of image artifacts (http://enigma.ini.usc.edu/protocols/dti-protocols/). In particular, the first b0 image was used as a reference for co-registration of the five subsequent b0 images (FSL FLIRT, Smith et al., 2004). The resulting co-registered b0 images were averaged and served as a reference image during motion and distortion correction on the diffusion-weighted images (Kreilkamp et al., 2015). In order to achieve distortion correction, the T2w image was rigidly aligned with the mean b0 image (Smith et al., 2004). The mean b0 image and the T2w image in diffusion space were thus combined to a pair of images with the first image representing geometric distortions while the T2w image served as a reference image without distortions. From this pair the susceptibility-induced off-resonance field was estimated (Andersson et al., 2003) and the two images were combined into a single corrected one, which was then brain-extracted. The diffusion-weighted images were corrected for motion and the resulting nonlinear registration information from the distortion correction step was used to unwarp subsequent diffusion-weighted images in native diffusion space (Andersson and Sotiropoulos, 2015). The gradient table information was adjusted according to the rigid body motion parameters (Leemans and Jones, 2009).

Macroscopic motion was assessed between groups using the Total Motion Index (TMI; Yendiki et al., 2013) to assess for confounding effects of motion on group-wise differences. Another possible confounding factor in DTI analysis are variations in signal-to-noise ratio (SNR), which has been shown to alter diffusion metrics (Farrell et al., 2007). We therefore wanted to rule out any effects of SNR on FA/MD group comparisons and performed a test on SNR values extracted from the motion corrected data (FSL eddy), which had subsequently been used during tensor estimation. Specifically, we used the AFQ WM segmentation map and thresholded it at 0.9, the resulting image was then used to mask the motion corrected diffusion-weighted images and extract mean and standard deviations for WM intensities to compute the ratio of the two (→ SNR), as proposed in Yendiki et al. (2011).

### 2.4 Manual and AFQ tractography

Manual tractography was performed in Diffusion Toolkit version 0.6.3 (http://www.trackvis.org) using the 2nd order Runge-Kutta tract propagation algorithm (step size of 1 mm, angle threshold of 35) in native diffusion space (Table 2). After tensor estimation and whole-brain tractography the UF and PHWM were analyzed. To achieve this, these two temporal lobe tracts were extracted with region-of-interest (ROI) placement on the native diffusion space FA image. Specifically, ‘AND’ ROIs were placed based on a previously published method to delineate fibers belonging to either UF or PHWM bundles, while ‘NOT’ ROIs eliminated fibers not belonging to these tracts (Wakana et al., 2007; Metzler-Baddeley et al., 2011). In particular, tracts of the inferior frontal-occipital fasciculus were removed when delineating the UF and projections to the occipital and contralateral lobes were removed when delineating the PHWM. For conventional whole tract analysis of FA and MD, tract density images were saved for bilateral UF and PHWM tracts and binarized.

**Table 2:** Commonalities and differences in manual and AFQ tractography steps.

Steps	Manual Tractography	AFQ
1. dMRI preprocessing	ENIGMA pipeline running FSL (identical)	
2. Conversion in space	none (native diffusion space)	conversion to T1w native space
3. Motion / eddy-current correction	none (performed in Step 1)	
4. Image resampling	none	resampling to isotropic 2 mm resolution with anatomical T1w alignment (7th order b-spline)
5. Tractography algorithm	2nd order Runge-Kutta	4th order Runge-Kutta
6. Tractography settings	deterministic, brain-masked, step size 1 mm, angle threshold 35°	
7. Removal of stray streamlines	based on Wakana et al. (2007)	
8. Conversion of tracts in space	none (apart from visualization in standard space with AFQ routines: Fig. 4, Fig. 5, Fig. 6)	tract volumes: back to native diffusion space via FSL FLIRT with 6 dof (whole tract analysis) tract streamlines: back to native diffusion space via along-tract-stats (only for visualizing in Fig. 1)
9. Calculations	native space for FA>0.2 (whole tract: mean; along-the-tract: weighted mean)	

AFQ = automated fiber quantification; dMRI = diffusion Magnetic Resonance Imaging; FA = fractional anisotropy; dof = Degrees of Freedom.

**Fig. 1:**
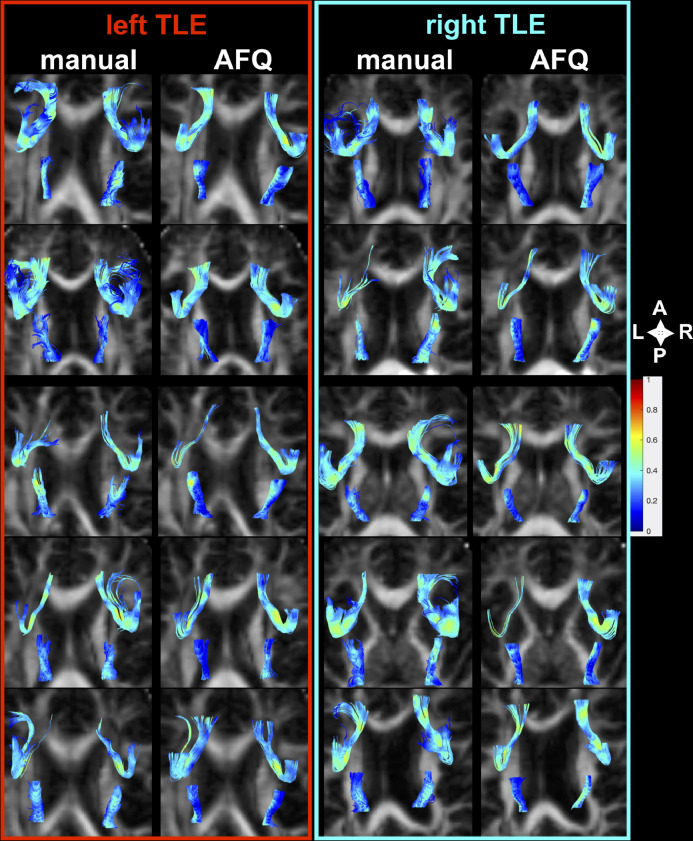
Manual and AFQ delineation of UF and PHWM tracts. Tract colors are defined by their FA values. The images with a bottom-up view were rendered in native diffusion space with along-tract-stats (Colby et al., 2012). *A*=anterior; *P*=posterior; *L*=left; *R*=right.

Automated whole-brain AFQ tractography was run using default parameters: forth-order Runge-Kutta approach (Yeatman et al., 2012; Table 2) based on the Euler method (step size of 1 mm, an angle threshold of 35; Basser et al., 2000). Prior to this AFQ performed a series of preprocessing steps automatically, including T1w co-registration for each diffusion-weighted image, brain extraction (Smith, 2002) and voxel-wise estimation of the diffusion tensor. After whole-brain tractography, AFQ non-linearly co-registered the mean b0 image to the International Consortium for Brain Mapping (ICBM) template and the inverse transformation was used to map the normalized ICBM WM ROIs provided within AFQ to the individual subject space. The ends of AFQ fiber bundles were defined by these ROIs and tracts were subsequently classified as belonging to the respective tracts. These steps were performed in individual T1w image space. Fig. 1 shows tract reconstructions in a random set of patients in both tractography approaches.

For conventional whole tract analysis of FA and MD, tract density images were saved for bilateral UF and PHWM tracts and binarized. After tract segmentation, tract cores for UF and PHWM were identified and FA/MD value profiles for each tract were calculated based on the weighted-mean approach, where FA/MD values of fibers close to the tract core contribute more to the along-the-tract measures than fibers distant to the core. This permitted tract profile group analysis within AFQ and manual tractography (Travis et al., 2015a; Yeatman et al., 2012; Keller et al., 2017). AFQ's weighted-mean approach was implemented into the along-tract-stats software (Colby et al., 2012) in order to analyze the manually delineated tracts in the same manner.

### 2.5 Statistical analysis

All data was non-normally distributed (Lilliefors Test *p*<0.05). Differences in demographic information between all three groups was assessed with a Kruskal-Wallis ANOVA (age) and chi-square test of independence (sex). An unpaired two-tailed Wilcoxon-Rank-Sum test was performed to assess differences in continuous clinical variables (age of onset, seizure burden, epilepsy duration corrected for age and seizure frequency per week) of left- and right-sided patients. A chi-square test of independence was used to analyze the effects of a history of infection, febrile seizures and secondary-generalized tonic-clonic seizures (SGTCS) on tract diffusion measures.

Significant group differences found between AFQ and manual tractography were directly compared. The manual tracts were truncated using the same ROI boundaries as those used in AFQ to allow for group-wise comparisons between the same regions along the tracts. Furthermore, FA maps were thresholded at FA>0.2, consistent with AFQ's thresholding (Yeatman et al., 2012). Whole tract unweighted mean FA and MD values were extracted from both tractography approaches using FSL software (Smith et al., 2004). All data was non-normally distributed, we therefore used the non-parametric Kruskal-Wallis ANOVA to investigate differences between participant groups (patients with left/right TLE and controls). Post-hoc analysis was conducted using the Bonferroni correction procedure. Furthermore, we assessed whole tract FA/MD value correlations between approaches using the Spearman correlation coefficient with Bonferroni multiple comparison correction. Tract FA/MD values were compared across manual and AFQ tractography approaches using the paired Wilcoxon-Signed-Rank test with post-hoc Bonferroni correction. The consistency in tract morphology of all clipped fibers generated by AFQ and the manual method were analyzed using the Dice coefficient (Dice, 1945). Results from Dice coefficient analysis indicate poor agreement (<0.2), fair agreement (0.2–0.4), moderate agreement (0.4–0.6), good agreement (0.6–0.8) and excellent agreement (0.8–1.00) (Dice, 1945). We additionally assessed gross volumes of automatically and manually generated tracts using a paired Wilcoxon-Signed-Rank test. Correlations between whole tract diffusion metrics and various clinical measures were investigated using the Spearman Rank Correlation Coefficient. These included seizure burden (log(duration of epilepsy x seizure frequency per week), age of onset, duration of epilepsy, duration of epilepsy corrected for age, seizure frequency per week and age. For every tractography approach, the Wilcoxon-Rank-Sum test was used to assess any differences between diffusion metrics and sex, the presence of HS, febrile seizures and SGTCS. All whole tract statistical results were corrected using the FDR procedure and results were considered significant at *p*<0.05.

AFQ and along-tract-stats (Colby et al., 2012) additionally allowed us to analyze diffusion metrics along the tracts among the two approaches and with respect to clinical features. Using this approach, comparisons were made between patients with left TLE, right TLE and controls. We also compared patients with HS to those without HS and controls. For this analysis, patients with right TLE were side-flipped and a subset of controls was also side-flipped to account for inter-hemispheric differences (Keller et al., 2015). Finally, demographic (age, sex) and clinical variables (age of onset, duration of epilepsy corrected for age, seizure burden and frequency, history of febrile and SGTC seizures) were correlated with diffusion characteristics as generated by AFQ along-the-tract analysis. Along-the-tract diffusion value and correlation analyses were corrected for multiple comparisons using the FDR procedure and results were considered significant at *p*<0.05.

## 3 Results

### 3.1 Demographic and clinical information

All demographic and clinical information can be found in Table 1. There were no significant group differences between any clinical or demographic variables.

### 3.2 Manual versus AFQ tractography

#### 3.2.1 Quality control

We found no significant differences in TMI between controls (Mean = 0.3; SD = 1.6), patients with left TLE (Mean = 0.4; SD = 1.4) and patients with right TLE (Mean = 0.02; SD =1.3); χ²(2,63) = 0.3, *p* = 0.9. The mean SNR for diffusion-weighted images did not significantly differ among groups: controls (Mean = 2.9; SD = 0.1), patients with left TLE (Mean = 2.8; SD = 0.1) and patients with right TLE (Mean = 2.8; SD = 0.2); χ²(2,63) = 4.1, *p* = 0.1.

#### 3.2.2 DTI-metrics

**Fig. 2:**
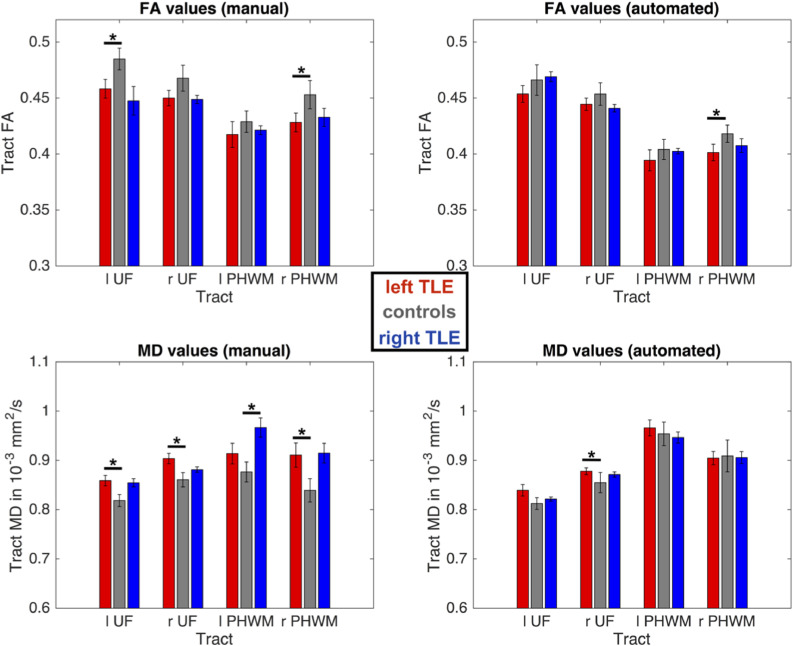
Whole tract diffusion measures for manual (left) versus AFQ (right) tractography. For each group and tract median FA/MD values are presented along with respective standard errors. Using manual tractography, patients with left TLE had significantly reduced FA values in the left UF and right PHWM and increased MD values in left and right UF and PHWM, while patients with right TLE had an increase in MD in the left PHWM compared to controls (top left and bottom left). AFQ was only able to identify the FA decrease in the right PHWM and MD increase in the right UF for patients with left TLE (bottom right). FA = Fractional Anisotropy; MD = Mean Diffusivity; UF = Uncinate Fasciculus; PHWM = Parahippocampal White Matter Bundle; *l* = left; *r* = right.

Whole WM tract FA/MD values across patient and control groups are shown in Fig. 2 and Table 3. Using AFQ, patients with left TLE had significantly reduced FA in the right PHWM and increased MD in the right UF relative to controls. These changes were also identified by the manual method including additional changes in patients relative to controls, reflected in decreased FA of the left UF and increased MD of left UF and right PHWM in left TLE and increased MD of left PHWM in right TLE.

**Table 3:** Whole tract diffusion measures for manual (top) versus AFQ (bottom) tractography.

		M (SD)			p-values (Bonferroni)			Statistics	
Tract Metric	Side	lTLE	C	rTLE	lTLE vs C	lTLE vs rTLE	C vs rTLE	χ²-statistic	p-value
Manual									
UF FA	l	0.46 (0.03)	0.48 (0.03)	0.45 (0.08)	<0.05	1	0.7	7.8	<0.05
	r	0.45 (0.03)	0.47 (0.03)	0.45 (0.02)	–	–	–	5	0.08
UF MD	l	0.86 (0.04)	0.82 (0.03)	0.85 (0.05)	<0.05	1	0.27	9.7	<0.01
	r	0.9 (0.04)	0.86 (0.04)	0.88 (0.03)	<0.05	1	0.85	8.6	<0.05
PHWM FA	l	0.42 (0.05)	0.43 (0.03)	0.42 (0.02)	–	–	–	1.9	0.4
	r	0.43 (0.04)	0.45 (0.04)	0.43 (0.05)	<0.05	1	1	6.8	<0.05
PHWM MD	l	0.9 (0.08)	0.88 (0.06)	0.97 (0.12)	0.4	0.8	<0.05	6.7	<0.05
	r	0.9 (0.1)	0.84 (0.07)	0.91 (0.1)	<0.05	1	0.26	8.7	<0.05
AFQ									
UF FA	l	0.45 (0.03)	0.47 (0.04)	0.47 (0.03)	–	–	–	3.7	0.2
	r	0.44 (0.02)	0.45 (0.03)	0.44 (0.02)	–	–	–	3.1	0.2
UF MD	l	0.84 (0.05)	0.82 (0.03)	0.81 (0.03)	–	–	–	5.2	0.07
	r	0.88 (0.03)	0.85 (0.06)	0.87 (0.03)	<0.05	1	0.7	6.4	<0.05
PHWM FA	l	0.39 (0.04)	0.4 (0.03)	0.4 (0.02)	–	–	–	1.3	0.5
	r	0.4 (0.03)	0.42 (0.02)	0.41 (0.04)	<0.05	0.69	1	7.02	<0.05
PHWM MD	l	0.97 (0.07)	0.95 (0.07)	0.95 (0.07)	–	–	–	1.2	0.5
	r	0.9 (0.05)	0.91 (0.1)	0.91 (0.1)	–	–	–	0.25	0.8

Abbreviations: *M* = Mean; SD = Standard Deviation; TLE = Temporal Lobe Epilepsy; *C* = Control; *l* = left; *r* = right; AFQ = Automated Fiber Quantification; FA = fractional anisotropy; MD = mean diffusivity (in 10^−3^ mm^2^/s); UF = uncinate fasciculus; PHWM = parahippocampal white matter bundle.

**Fig. 3:**
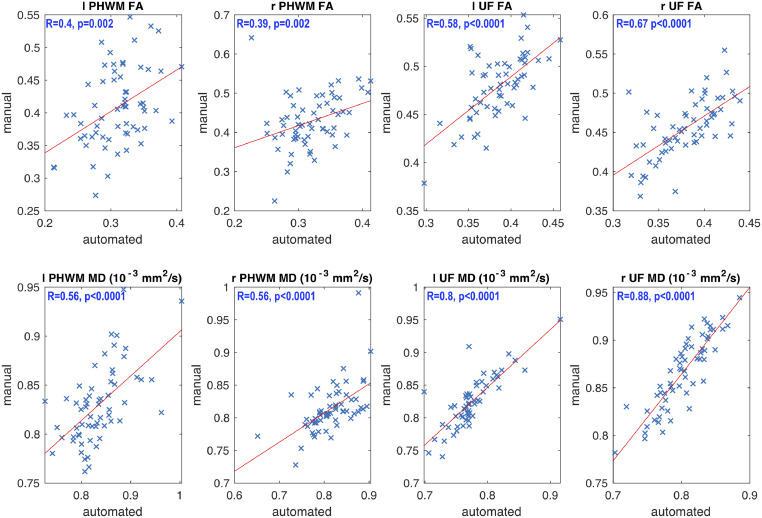
Correlations of whole tract diffusion measures. There were significant correlations between all extracted FA and PHWM MD values extracted by manual and AFQ tractography and least square lines were fitted to these plots. FA = Fractional Anisotropy; MD = Mean Diffusivity; UF = Uncinate Fasciculus; PHWM = Parahippocampal White Matter Bundle; *l* = left; *r* = right; *R* = Spearman's Rho.

**Fig. 4:**
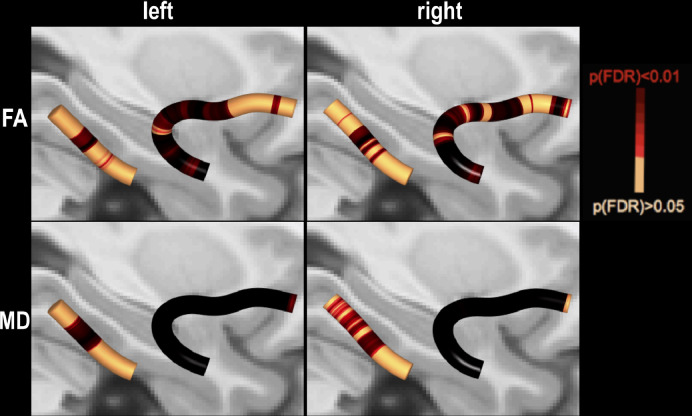
Correlations of along-the-tract diffusion measures. There were significant correlations between all extracted FA/MD values extracted by manual and AFQ tractography. FA = Fractional Anisotropy; MD = Mean Diffusivity.

FA and MD values extracted from the two different approaches were significantly correlated for each tract. All correlations survived Bonferroni correction; *p*(corr) = *α/n* = 0.05/8 = 0.006, Fig. 3. A comparison between manual and AFQ tractography FA and MD values revealed a significant difference only for the FA value in the left UF. However, AFQ computed significantly lower FA and higher MD values for the bilateral PHWM and lower FA for the left UF relative to the manual tractography approach (Table 4). Along-the-tract correlation analysis revealed significant correlations between the two approaches for all tracts (Fig. 4).

**Table 4:** Comparison of FA/MD values from all tracts between tractography approaches.

	UF FA		UF MD (in 10^–3^mm^2^/s)		PHWM FA		PHWM MD (in 10^–3^mm^2^/s)	
	left	right	left	right	left	right	left	right
Manual	0.47 (0.04)	0.46 (0.03)	0.83 (0.04)	0.87 (0.04)	0.43 (0.3)	0.44 (0.04)	0.9 (0.08)	0.87 (0.09)
AFQ	0.46 (0.04)	0.45 (0.03)	0.82 (0.04)	0.86 (0.05)	0.4 (0.03)	0.41 (0.03)	0.96 (0.07)	0.91 (0.08)
*Z; p*	2.1; 0.03	1.8; 0.07	1.9; 0.05	1.8; 0.07	4.4; <0.001	5.1; <0.001	-4.7; <0.001	-3.7; <0.001

Mean and standard deviations (in brackets) are presented for each tract along with the results of the Wilcoxon-Rank-Sum test. After Bonferroni Correction (*p*(corr) = *α/n* = 0.05/8 = 0.006), no significant effects were observed for UF values. AFQ computed lower FA and higher MD values for PHWM compared to manual tractography. UF = uncinate fasciculus; PHWM = parahippocampal white matter bundle; FA = fractional anisotropy; MD = mean diffusivity.

#### 3.2.3 Consistency of tracts

The automatically reconstructed tract bundles had increased volume (Mean = 2570; SD = 1108) when compared to manual tracts (Mean = 1006; SD = 567) due to a higher number of streamlines; paired Wilcoxon-Signed-Rank test (*Z* = 13.6; *p*<0.001). The Dice Coefficient results for tract bundles were 0.54 (+/-0.12) for the left UF, 0.57 (+/-0.14) for the right UF, 0.6 (+/-0.12) for the right PHWM and 0.6 (+/-0.08) for the left PHWM. The agreement between the two methods was moderate for UF (bilateral) and good for PHWM (bilateral).

#### 3.2.4 Correlations between whole tract DTI metrics and clinical variables

No correlations between clinical variables and DTI-metrics extracted through manual tractography (Table 5) were found. However, in AFQ there were significant correlations between younger age of onset and decreased ipsilateral PHWM FA (*R* = 0.6; p_(FDR)_<0.05) and increased MD (*R* = -0.6; p_(FDR)_<0.05) and longer duration of epilepsy and increased ipsilateral PHWM MD (*R* = 0.8; p_(FDR)_<0.01), surviving correction for age (*R* = 0.7; p_(FDR)_<0.01). There were no significant effects of sex, HS, SGTCS or history of febrile seizures on diffusion scalar metrics (Table 6).

**Table 5:** Correlations of whole tract FA/MD values with variables for both approaches.

		Manual Tractography						AFQ Tractography					
Tract Metric	Side	Age	Age of Onset	Burden	Duration	Duration (corr. Age)	Frequency	Age	Age of Onset	Burden	Duration	Duration (corr. Age)	Frequency
UF FA	ipsi	*R*=-0.6	*R* = 0.01	*R*=-0.4	*R*=-0.4	*R*=-0.3	*R*=-0.2	*R*=-0.4	*R* = 0.01	*R*=-0.1	*R*=-0.2	*R*=-0.1	*R*=-0.1
		*p* = 0.1	*p* = 1	*p* = 0.3	*p* = 0.2	*p* = 0.6	*p* = 0.7	*p* = 0.3	*p* = 1	*p* = 1	*p* = 0.6	*p* = 0.8	*p* = 0.8
	contra	*R* = 0.1	*R* = 0.5	*R*=-0.1	*R*=-0.3	*R*=-0.4	*R* = 0.2	*R*=-0.2	*R* = 0.3	*R*=-0.3	*R*=-0.5	*R*=-0.4	*R* = 0.1
		*p* = 0.8	*p* = 0.2	*p* = 1	*p* = 0.4	*p* = 0.2	*p* = 0.7	*p* = 0.5	*p* = 0.4	*p* = 0.5	*p* = 0.1	*p* = 0.2	*p* = 1
UF MD	ipsi	*R* = 0.3	*R*=-0.1	*R*=-0.1	*R* = 0.3	*R* = 0.2	*R*=-0.2	*R* = 0.1	*R*=-0.3	*R*=-0.1	*R* = 0.2	*R* = 0.3	*R*=-0.2
		*p* = 0.4	*p* = 0.9	*p* = 1	*p* = 0.5	*p* = 0.7	*p* = 0.6	*p* = 0.8	*p* = 0.5	*p* = 0.9	*p* = 0.5	*p* = 0.5	*p* = 0.5
	contra	*R* = 0.01	*R*=-0.5	*R* = 0.2	*R* = 0.5	*R* = 0.6	*R* = 0.01	*R* = 0.1	*R*=-0.4	*R* = 0.3	*R* = 0.5	*R* = 0.5	*R* = 0.1
		*p* = 1	*p* = 0.1	*p* = 0.7	*p* = 0.1	*p* = 0.1	*p* = 1	*p* = 0.9	*p* = 0.3	*p* = 0.4	*p* = 0.1	*p* = 0.1	*p* = 0.8
PHWM FA	ipsi	*R*=-0.03	*R* = 0.6	*R*=-0.2	*R*=-0.4	*R*=-0.5	*R* = 0.02	*R*=-0.1	**R** = **0.6**	*R*=-0.2	*R*=-0.5	*R*=-0.6	*R*=-0.02
		*p* = 1	*p* = 0.1	*p* = 0.7	*p* = 0.2	*p* = 0.1	*p* = 1	*p* = 0.8	***p*** = **0.02**	*p* = 0.5	*p* = 0.1	*p* = 0.1	*p* = 1
	contra	*R*=-0.1	*R* = 0.4	*R*=-0.01	*R*=-0.3	*R*=-0.3	*R* = 0.02	*R*=-0.02	*R* = 0.4	*R* = 0.01	*R*=-0.4	*R*=-0.4	*R* = 0.1
		*p* = 1	*p* = 0.2	*p* = 1	*p* = 0.4	*p* = 0.4	*p* = 1	*p* = 1	*p* = 0.2	*p* = 1	*p* = 0.3	*p* = 0.3	*p* = 0.9
PHWM MD	ipsi	*R* = 0.4	*R*=-0.3	*R* = 0.1	*R* = 0.5	*R* = 0.4	*R*=-0.2	*R* = 0.3	***R***=-**0.6**	*R* = 0.3	***R*** = **0.8**	***R*** = **0.7**	*R*=-0.02
		*p* = 0.2	*p* = 0.5	*p* = 1	*p* = 0.2	*p* = 0.2	*p* = 0.6	*p* = 0.3	***p*** = **0.02**	*p* = 0.3	***p***<**0.001**	***p***<**0.01**	*p* = 1
	contra	*R* = 0.4	*R* = 0.02	*R*=-0.1	*R* = 0.3	*R* = 0.2	*R*=-0.2	*R* = 0.3	*R*=-0.1	*R* = 0.2	*R* = 0.4	*R* = 0.3	*R* = 0.01
		*p* = 0.2	*p* = 1	*p* = 1	*p* = 0.5	*p* = 0.7	*p* = 0.6	*p* = 0.3	*p* = 0.9	*p* = 0.6	*p* = 0.2	*p* = 0.5	*p* = 1

Spearman rho values (R) are shown with FDR corrected p-values (p) for each type of analysis (manual and automated). Boldface indicates significant effects. UF = Uncinate Fasciculus; PHWM = Parahippocampal White Matter Bundle; FA = Fractional Anisotropy; MD = Mean Diffusivity; ipsi = ipsilateral; contra = contralateral.

**Table 6:** Comparison of FA/MD values from all tracts between patient groups according to sex, presence of HS, SGTCS and history of febrile seizures.

	UF FA		UF MD (in 10^–3^mm^2^/s)		PHWM FA		PHWM MD (in 10^–3^mm^2^/s)	
	Ipsi	Contra	Ipsi	Contra	Ipsi	Contra	Ipsi	Contra
MANUAL								
female	0.45 (0.03)	0.44 (0.05)	0.87 (0.05)	0.89 (0.06)	0.4 (0.04)	0.42 (0.02)	0.92 (0.08)	0.92 (0.09)
male	0.46 (0.03)	0.46 (0.03)	0.86 (0.03)	0.89 (0.04)	0.4 (0.05)	0.44 (0.04)	0.9 (0.13)	0.94 (0.14)
*Z; p(FDR)*	0; 1	-0.8; 0.8	0.5; 1	-0.4; 1	-1.3; 0.67	-1.2; 0.68	1.1; 0.69	0.15; 1
SGTCS	0.46 (0.04)	0.45 (0.06)	0.87 (0.05)	0.88 (0.04)	0.44 (0.05)	0.43 (0.03)	0.89 (0.07)	0.91 (0.08)
SGTCS No	0.46 (0.01)	0.44 (0.02)	0.85 (0.03)	0.91 (0.06)	0.41 (0.04)	0.42 (0.03)	0.95 (0.12)	0.96 (0.14)
*Z; p(FDR)*	0.5; 1	1.4; 0.67	0.96; 0.78	-1.6; 0.67	1.67; 0.67	1.67; 0.67	-1.46; 0.67	-0.76; 0.84
HS	0.45 (0.04)	0.45 (0.03)	0.88 (0.05)	0.89 (0.05)	0.41 (0.05)	0.42 (0.04)	0.95 (0.08)	0.92 (0.08)
HS No	0.46 (0.02)	0.45 (0.05)	0.86 (0.04)	0.89 (0.05)	0.43 (0.05)	0.43 (0.03)	0.9 (0.1)	0.93 (0.12)
*Z; p(FDR)*	-0.2; 1	-0.03; 1	0.6; 0.98	0.03; 1	-1.2; 0.68	-0.2; 1	1.6; 0.67	0.18; 1
Febrile	0.45 (0.04)	0.45 (0.03)	0.87 (0.05)	0.89 (0.06)	0.39 (0.04)	0.41 (0.03)	0.97 (0.07)	0.94 (0.07)
Febrile No	0.46 (0.03)	0.45 (0.05)	0.86 (0.04)	0.89 (0.05)	0.43 (0.05)	0.43 (0.03)	0.9 (0.1)	0.93 (0.1)
*Z; p(FDR)*	0.16; 1	-0.3; 1	0.16; 1	0; 1	-1.4; 0.67	-1; 0.78	1.8; 0.67	0.8; 0.8
AFQ								
female	0.44 (0.02)	0.45 (0.02)	0.86 (0.05)	0.85 (0.04)	0.39 (0.04)	0.39 (0.01)	0.95 (0.08)	0.91 (0.05)
male	0.47 (0.03)	0.46 (0.04)	0.85 (0.03)	0.87 (0.05)	0.42 (0.03)	0.42 (0.04)	0.93 (0.06)	0.92 (0.09)
*Z; p(FDR)*	-2.2; 0.6	-0.1; 1	0.2; 1	-0.84; 0.87	-1.6; 0.63	-1.6; 0.63	0.34; 1	0.03; 1
SGTCS	0.45 (0.03)	0.46 (0.03)	0.86 (0.05)	0.85 (0.04)	0.4 (0.04)	0.41 (0.03)	0.94 (0.08)	0.91 (0.06)
SGTCS No	0.45 (0.01)	0.45 (0.02)	0.83 (0.02)	0.87 (0.04)	0.4 (0.03)	0.4 (0.02)	0.95 (0.07)	0.9 (0.07)
*Z; p(FDR)*	-0.65; 0.87	0.47; 0.97	1.4; 0.7	-0.79; 0.87	0.3; 1	1; 0.87	-0.2; 1	-1; 0.87
HS	0.44 (0.04)	0.45 (0.03)	0.87 (0.06)	0.87 (0.03)	0.38 (0.04)	0.39 (0.02)	0.99 (0.08)	0.92 (0.05)
HS No	0.45 (0.02)	0.45 (0.03)	0.84 (0.03)	0.86 (0.04)	0.41 (0.03)	0.41 (0.03)	0.92 (0.06)	0.92 (0.07)
*Z; p(FDR)*	-0.7; 0.87	-0.7; 0.87	0.67; 0.87	0.5; 0.97	-1.87; 0.6	-0.77; 0.87	1.7; 0.63	0.03; 1
Febrile	0.44 (0.03)	0.44 (0.01)	0.86 (0.06)	0.86 (0.04)	0.37 (0.04)	0.39 (0.02)	0.97 (0.1)	0.92 (0.05)
Febrile No	0.45 (0.03)	0.46 (0.03)	0.85 (0.04)	0.86 (0.04)	0.41 (0.04)	0.41 (0.03)	0.94 (0.6)	0.92 (0.07)
*Z; p(FDR)*	-0.3; 1	-1.1; 0.87	0; 1	0; 1	-1.6; 0.63	-1.4; 0.7	0.7; 0.87	0.14; 1

Mean and standard deviations (in brackets) are presented for each tract. No significant effects were observed for either manual or AFQ generated tracts. UF = uncinate fasciculus; PHWM = parahippocampal white matter bundle; FA = fractional anisotropy; MD = mean diffusivity; ipsi = ipsilateral; contra = contralateral; HS = hippocampal sclerosis; SGTCS = secondary-generalized tonic-clonic seizures.

### 3.3 Along-the-tract analysis

**Fig. 5:**
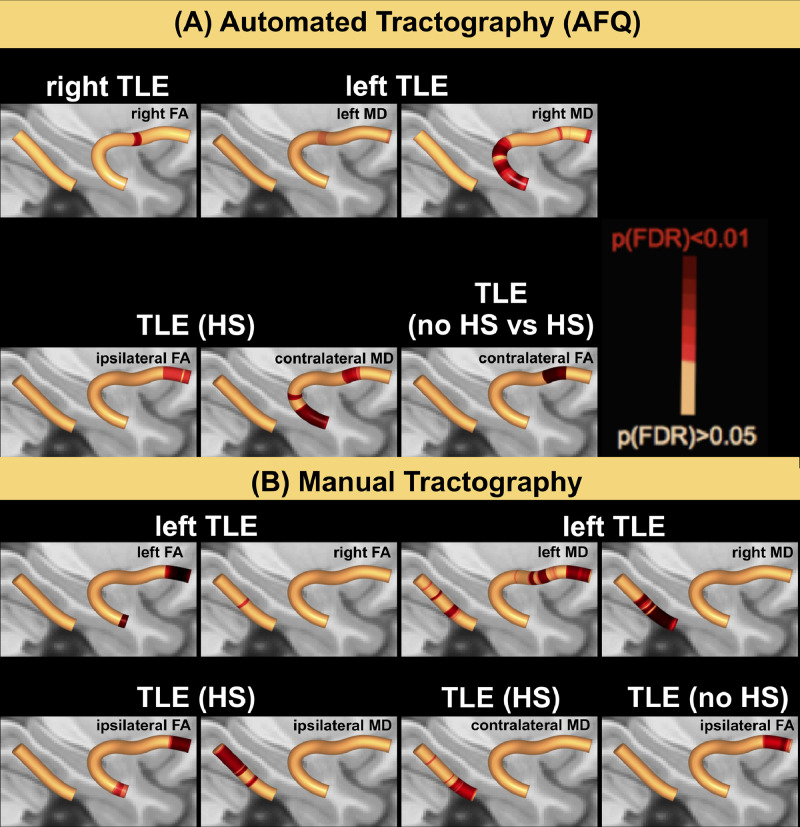
Comparison of patient and control groups. The T1w overlay in standard space shows areas of the UF where patients had decreased FA (left, red areas) and increased MD (right and inset, red areas) in both automated (A) and manual (B) approaches. rTLE = right TLE; lTLE = left TLE; FA = fractional anisotropy; MD = mean diffusivity; HS = Hippocampal Sclerosis.

Using AFQ we found significantly reduced FA in a small area of the right UF in patients with right TLE relative to controls, while patients with left TLE had significantly increased MD in two small regions of the left UF and in large portions of the right UF relative to controls (Fig. 5, Panel A). When performing manual tractography we did not find any diffusivity changes in patients with right TLE compared to controls. However, we found significantly decreased FA in the left UF and right PHWM, and an increased MD in left and right PHWM and left anterior UF (Fig. 5, Panel B).

Furthermore, we tested patients with and without HS against controls. There was significantly decreased FA in the frontal part of the ipsilateral UF and significantly increased MD in the anterior and temporal parts of the contralateral UF in patients with HS compared to controls (Fig. 5, Panel A). For the manual approach, we found significantly decreased FA in the anterior and temporal parts of the ipsilateral UF and increased MD in the ipsi- and contralateral PHWM (Fig. 5, Panel B). Across all investigated tracts, there were no significant differences between patients without HS and controls when using the AFQ. However, using the manual approach, we found decreased FA in the ipsilateral anterior UF in this group when comparing them to controls. For AFQ, patients with HS had significantly decreased FA in the frontal part of the contralateral UF relative to patients without HS (Fig. 5, Panel A).

**Fig. 6:**
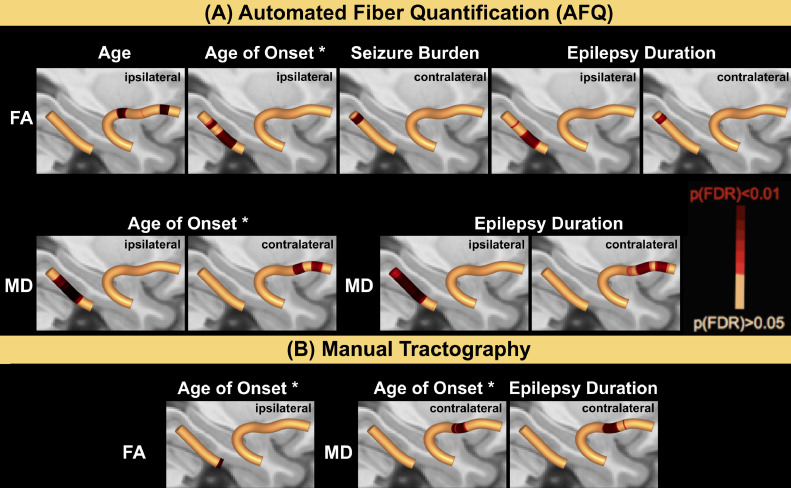
Correlations of DTI-metrics with demographic and clinical variables. There were correlations between FA and age, age of onset, seizure burden and epilepsy duration corrected for age. All FA correlations were negative except for the correlation with age of onset, which was positive (marked with an asterisk). There were also correlations between patient MD values and age of onset (negative, marked with an asterisk) and epilepsy duration corrected for age (positive). These changes were present in automated (A) and manual tractography (B). FA = fractional anisotropy; MD = mean diffusivity.

For all patients we investigated correlations between along-the-tract FA/MD extracted via AFQ with demographic and clinical variables (Fig. 6, Panel A). Several significant correlations were found:

(i) age (ipsilateral decrease of FA in UF with increasing age),
(ii) younger age at onset (decrease of FA in ipsilateral PHWM; increase of MD in ipsilateral PHWM and contralateral UF),
(iii) increased seizure burden correlated with a decrease in FA in a posterior section of the contralateral PHWM,
(iv) a longer epilepsy duration corrected for age correlated with a decrease of FA in ipsilateral and contralateral PHWM,
(v) an increase of MD in ipsilateral PHWM and contralateral UF.

In manual tractography, a younger age of onset was correlated with decreased FA values of the ipsilateral PHWM and increased MD values within the contralateral UF. Duration of epilepsy corrected for age correlated positively with MD values found in the contralateral UF (Fig. 6, Panel B).

## 4 Discussion

The objective of this study was to determine the agreement between manually and automatically generated temporal lobe WM tracts in patients with TLE. We sought to investigate the consistency between the automated and manual approaches with respect to (i) tract morphological characteristics using Dice Coefficient analyses, (ii) similarities in diffusivity scalar metrics (FA and MD) and (iii) the sensitivity of each approach for the detection of tract diffusivity abnormalities in patients relative to controls and correlations between clinical characteristics and diffusion metrics.

Automated and manual tractography revealed a moderate to good Dice Coefficient and a strong correlation between whole and along-tract FA/MD values. Nevertheless, AFQ estimated significantly lower FA and higher MD values in the PHWM tract. The manual approach was more sensitive than AFQ in identifying whole tract diffusion changes in patients relative to controls. No correlations between manual whole tract diffusion characteristics and clinical variables survived correction for multiple comparisons. Conversely AFQ's whole tract metrics revealed correlations with clinical variables such as age of onset and duration of epilepsy corrected for age. These were also confirmed through along-the-tract analysis within AFQ and manual tractography. For both tractography approaches, patients with left TLE showed more diffusivity alterations than patients with right TLE when either group was compared to controls. We highlight methodological issues of this study before discussing the biological implications in light of the current literature.

### 4.1 Strengths and limitations

The direct comparison of manual and AFQ tractography approaches revealed that tracts and extracted diffusion values by these two approaches correspond to each other. However, minor differences were found (i) in tract volume, where AFQ showed increased volume relative to the manual approach (ii) in group whole tract analysis where AFQ did not identify as many tracts with abnormal diffusivity values in patients with left TLE when compared to the manual approach and (iii) the whole tract correlation analysis with clinical variables only showed significant results when using AFQ relative to the manual approach, but along-the-tract analysis revealed significant clinical correlations (age of onset and duration) in both approaches. It is likely that for (i) the difference in tract volume may have originated from the fact that only the 2nd order Runge-Kutta algorithm without previous co-registration to T1w and voxel resampling was available for the manual approach, while for AFQ the default tractography algorithm had been set at the 4th order Runge-Kutta algorithm together with previous upsampling to isotropic 1 mm T1w space and subsequent downsampling to isotropic 2 mm resolution by the developers (Yeatman et al., 2012). As diffusion metrics were thus sampled from a larger tract volume within AFQ, this may explain why whole tract diffusion alterations present in the manual approach were not detected with AFQ in patients with left TLE (ii). However, when investigating correlations between whole tract diffusion metrics and clinical variables, these were only detected when using AFQ (iii). This may be the result of the higher fiber volume found in AFQ and consequently the inclusion of DTI values from white matter tractography streamlines that are more distant from the fiber's core. Clinical correlations found in whole tract AFQ analysis were replicated using along-the-tract analysis within AFQ and the manual approach, which then revealed the localized correlations of age of onset and duration with diffusivity metrics. Some group results identified using the manual tractography approach were not identified by AFQ. This may be explained by the fact that there was less across-group data variance when analyzing tracts using AFQ due to the pre-defined tract-segmentation ROIs applied from standard space to T1w space. Ultimately, studies aiming to compare tractography approaches in more detail should harmonize identical tractography algorithms to run in native diffusion space so that stronger conclusions may be justified and the automated approach with the closest correspondence to neuroanatomy can be identified. The combination of diffusion and histology analysis (Concha et al., 2010), which was not possible in our study due to lack of histological data from study participants, may be able to resolve additional research questions with respect to biological implications such as loss or disruption of myelin and axons.

In order to investigate the relationship between DTI-metrics and clinical variables and group diffusivity alterations across patients with and without HS and controls, the information obtained for each tract had to be side-flipped in patients with right TLE and the corresponding subset of controls. This is a common procedure and often necessary, as the prevalence of right TLE is lower than that of left TLE (Giovagnoli, 2001; Manaut et al., 2002; Njiokiktjien, 2005). Although side-flipping has been performed in many previous studies aiming to assess correlations and group-wise differences in small sample sizes (Keller et al., 2015; Coan et al., 2014; Imamura et al., 2015; Müller et al., 2006; Fonseca et al., 2012), one limitation of this is that the analysis does not allow judgements on diffusivity alterations that are dependent on right- or left-sided seizure onsets.

### 4.2 Biological implications

In contrast to patients with right TLE, those with left TLE showed more extensive and severe ipsi- and contralateral WM disruption (consistent with: Ahmadi et al., 2009, Kreilkamp et al., 2017, Keller et al., 2012b, Kemmotsu et al., 2011). Concerning patients with right TLE, apart from an increase in MD in the left PHWM using manual tractography, no diffusion alterations were detected. Brain damage in early childhood predominates in the left hemisphere and the left-right difference is also present in patients with unilateral TLE (Njiokiktjien, 2005). It is likely that structures within the left hemisphere are more vulnerable to initial precipitating injuries than the right (Keller et al., 2012b; Kemmotsu et al., 2011; Miro et al., 2015). The left-right maturational gradient had been described by Corballis and Morgan (1978) as an earlier and more rapid development of the left hemisphere compared to the right, which may suggest an early critical period for left hemispheric development. More detailed longitudinal studies are necessary to allow statements on neurodevelopmental factors influencing hemispheric vulnerability and to investigate whether left and right TLE are etiologically and pathologically distinct subtypes of TLE (Ahmadi et al., 2009).

Concha et al. (2009), Liu et al. (2012), Campos et al. (2015) and this present study have found that patients with TLE and associated HS had more widespread diffusion alterations than patients without HS. This may indicate differential epileptogenic networks involved in lesional and non-lesional TLE (Liu et al., 2012) and that a pathologic hippocampus can influence WM tract integrity on the contralateral hemisphere, a finding that is consistent with other studies discussing patients with left TLE and HS (Kreilkamp et al., 2017; Keller et al., 2012b; Kemmotsu et al., 2011; Keller et al., 2002; Ahmadi et al., 2009; Bonilha et al., 2007). However, it should be noted, that other studies have reported this for WM (Liu et al., 2012) and GM (Garcia-Fiñana et al., 2006; Pail et al., 2010) in patients with right TLE and HS. A younger age of onset was associated with decreased FA along the ipsilateral PHWM tract, which was also found by Kemmotsu et al. (2011) in patients with left TLE. Furthermore, these authors could identify a significant relationship between decreased FA of the ipsilateral/contralateral UF and age of onset, while we found a negative correlation between MD of the ipsilateral PHWM and contralateral UF with age of onset (manual and AFQ tractography). These correlations may be expected, as an early onset of epilepsy has been linked to decreased cognitive functioning (Elger et al., 2004) and unfavorable post-operative outcomes (Kwan and Brodie, 2000). Using AFQ, we found a negative correlation with epilepsy duration with FA in the ipsilateral and contralateral PHWM, while MD in the ipsilateral PHWM and contralateral UF correlated positively with epilepsy duration corrected for age (also present in manual tractography). The negative correlation between duration corrected for age and FA of ipsilateral (Keller et al., 2012b) and contralateral temporal lobe WM (Kemmotsu et al., 2011) has been previously identified. Some other studies have not been able to determine correlations of diffusivity alterations with clinical variables (Keller et al., 2002; Thivard et al., 2005) due to small sample sizes (Keller et al., 2012b). Performing conservative statistical testing taking non-normal distribution of the data and multiple testing into account as performed within the present study (and similarly in Garcia-Finana et al., 2006) or not may also influence reports on significant correlations.

### 4.3 Conclusion

The main objective of this study was to compare the manual and AFQ approaches in analysis of patients with TLE. One of the major strengths when analyzing data via AFQ tractography, which performed similarly relative to time-consuming manual tractography, is the possibility of time-efficient reproducibility, detailed quality assurance and documented control giving researchers and clinicians a ready-to-use and sensitive tool. The present study using AFQ was able to corroborate previous results found when patients with left/right TLE and patients with/without HS were compared to controls and when clinical correlations were assessed. Given the absence of excellent agreement between manual technique and AFQ, caution should be considered when using AFQ tractography particularly when used without reference to benchmark measures. Future studies should aim to perform comparisons of automated and manual tractography approaches via the same tractography algorithm implemented into harmonized protocols. Eventually, this will also aid larger multi-site research projects (such as ENIGMA-Epilepsy, http://enigma.ini.usc.edu/ongoing/enigma-epilepsy/) and the community to develop automated WM tract analysis for individual patients.
